# Skeletal muscle mechanics, energetics and plasticity

**DOI:** 10.1186/s12984-017-0318-y

**Published:** 2017-10-23

**Authors:** Richard L. Lieber, Thomas J. Roberts, Silvia S. Blemker, Sabrina S. M. Lee, Walter Herzog

**Affiliations:** 1 Rehabilitation Institute of Chicago, Chicago, USA; 2 Brown University, Providence, USA; 3 University of Virginia, Charlottesville, USA; 4 Northwestern University, Evanston, USA; 5 University of Calgary, Faculty of Kinesiology, Calgary, Canada

**Keywords:** Muscle mechanics, Cross-bridge theory, Sarcomeres, Residual force enhancement, Muscle modeling, Force sharing, Sliding filament, Titin

## Abstract

The following papers by Richard Lieber (Skeletal Muscle as an Actuator), Thomas Roberts (Elastic Mechanisms and Muscle Function), Silvia Blemker (Skeletal Muscle has a Mind of its Own: a Computational Framework to Model the Complex Process of Muscle Adaptation) and Sabrina Lee (Muscle Properties of Spastic Muscle (Stroke and CP) are summaries of their representative contributions for the session on skeletal muscle mechanics, energetics and plasticity at the 2016 Biomechanics and Neural Control of Movement Conference (BANCOM 2016). Dr. Lieber revisits the topic of sarcomere length as a fundamental property of skeletal muscle contraction. Specifically, problems associated with sarcomere length non-uniformity and the role of sarcomerogenesis in diseases such as cerebral palsy are critically discussed. Dr. Roberts then makes us aware of the (often neglected) role of the passive tissues in muscles and discusses the properties of parallel elasticity and series elasticity, and their role in muscle function. Specifically, he identifies the merits of analyzing muscle deformations in three dimensions (rather than just two), because of the potential decoupling of the parallel elastic element length from the contractile element length, and reviews the associated implications for the architectural gear ratio of skeletal muscle contraction. Dr. Blemker then tackles muscle adaptation using a novel way of looking at adaptive processes and what might drive adaptation. She argues that cells do not have pre-programmed behaviors that are controlled by the nervous system. Rather, the adaptive responses of muscle fibers are determined by sub-cellular signaling pathways that are affected by mechanical and biochemical stimuli; an exciting framework with lots of potential. Finally, Dr. Lee takes on the challenging task of determining human muscle properties in vivo. She identifies the dilemma of how we can demonstrate the effectiveness of a treatment, specifically in cases of muscle spasticity following stroke or in children with cerebral palsy. She then discusses the merits of ultrasound based elastography, and the clinical possibilities this technique might hold. Overall, we are treated to a vast array of basic and clinical problems in skeletal muscle mechanics and physiology, with some solutions, and many suggestions for future research.

## Background

On June 12–16, 2016, approximately 150 scientists in the areas of biomechanics and neural control of movement met at the Deer Creek Lodge in Sterling Ohio for an unusual meeting. The meeting was unusual since it only had happened once before, 20 years earlier, and it was unusual because half of the available time was set aside for discussion, a format that allowed for significant contributions by the attendees.

I was invited to this conference with the mandate to chair a session on skeletal muscle mechanics, energetics and plasticity. This was an exciting prospect as the speakers and topics for that session had already been identified: Drs. Rick Lieber (Skeletal Muscle as an Actuator), Tom Roberts (Elastic Mechanisms and Muscle Function), Silvia Blemker (Skeletal Muscle has a Mind of its Own: a Computational Framework to Model the Complex Process of Muscle Adaptation) and Sabrina Lee (Muscle Properties of Spastic Muscle (Stroke and CP). Dr. Lieber revisits the topic of sarcomere length as a fundamental property of skeletal muscle contraction. Specifically, problems associated with sarcomere length non-uniformity and the role of sarcomerogenesis in diseases such as cerebral palsy are critically discussed. Dr. Roberts then makes us aware of the (often neglected) role of the passive tissues in muscles and discusses the properties of parallel elasticity and series elasticity, and their role in muscle function. Specifically, he identifies the merits of analyzing muscle deformations in three dimensions (rather than just two), because of the potential decoupling of the parallel elastic element length from the contractile element length, and reviews the associated implications for the architectural gear ratio of skeletal muscle contraction. Dr. Blemker tackles muscle adaptation using a novel way of looking at adaptive processes and what might drive adaptation. She argues that cells do not have pre-programmed behaviors that are controlled by the nervous system. Rather, the adaptive responses of muscle fibers are determined by sub-cellular signaling pathways that are affected by mechanical and biochemical stimuli; an exciting framework with lots of potential. Finally, Dr. Lee takes on the challenging task of determining human muscle properties in vivo. She identifies the dilemma of how we can demonstrate the effectiveness of a treatment, specifically in cases of muscle spasticity following stroke or in children with cerebral palsy. She then discusses the merits of ultrasound based elastography, and the clinical possibilities this technique might hold. Indeed a powerful group of individuals with broad backgrounds in skeletal muscle mechanics, physiology, structure and function.

Despite the apparently different topics, they all are focused on the mechanics, properties and function of in vivo human skeletal muscles. Drs. Lieber and Roberts discuss the importance of sarcomere properties, sarcomere non-uniformities and elasticity for the everyday function of muscles. This topic is then expanded by Dr. Lee who introduces new technologies to assess muscle properties in vivo, and Dr. Blemker provides a theoretical framework not only for muscle properties and muscle function, but also for adaptive processes in skeletal muscle training, adaptation, disease, and aging. All four contributions are ultimately aimed at understanding how muscles work in the animal body, and how muscle properties and function adapt to external challenges.

There had been a previous BANCOM meeting, 20 years earlier, with many of the most prominent scientists of the time. Some of them have retired in the meantime, others returned to this second installment. BANCOM 1996 represented a state of the art assessment of the knowledge in the area of muscle function and movement control. Questions were raised that have been solved in the meantime, while others are still wide open. The 2016 installment of the BANCOM conference was an equally exciting gathering. Critical research areas and questions to be answered were discussed, and critical and constructive discussions continued beyond the official meeting schedule. The contributions by Drs. Lieber, Roberts, Blemker and Lee, represent a state-of-the-art understanding of current in vivo muscle mechanics, they raise a series of questions and propose challenges. It will be interesting to compare our understanding of these issues today, with our understanding in 20 years from now, when hopefully, somebody will organize the 3rd gathering of BANCOM researchers.

## Skeletal muscle as an actuator

**Fig. 1:**
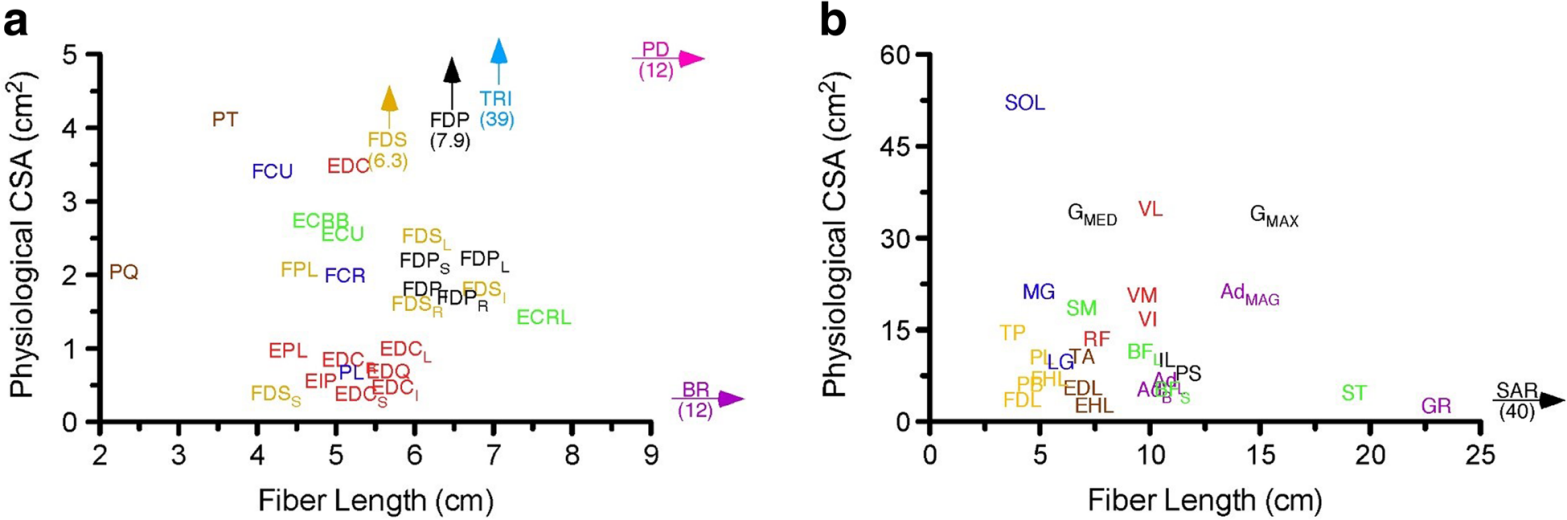
**a** Scatter graph of fiber length and physiological cross-sectional areas of muscles in the human upper limb. Fiber length is proportional to muscle excursion while physiological cross-sectional area is proportional to maximum muscle force. Thus, this graph can be used to compare the relative forces and excursions of arm and forearm muscles. (Data and abbreviations from: [2]). **b** Scatter graph of fiber length and physiological cross-sectional areas of muscles in the human lower limb. Fiber length is proportional to muscle excursion while physiological cross-sectional area is proportional to maximum muscle force. Thus, this graph can be used to compare the relative forces and excursions of leg muscles. Muscles placed at extremes of graph (SOL, GR, and SAR) would be plotted off this scale at the position shown. (Data and abbreviations from: [7])

Throughout the BANCOM meeting, skeletal muscle has been portrayed as an actuator with fairly well defined properties. In this section, we present an overview of the basic properties of skeletal muscles as well as their ability to adapt. For some time now, the architecture of skeletal muscle, which is defined as the orientation and number of fibers within a muscle, has been considered the gold standard for a first pass definition of muscle problems [1, 2]. The two most important skeletal muscle properties are the length of the muscle fibers (which determines muscle excursion) and physiological cross sectional area (PCSA; which determine the peak muscle force). Validation of this statement has primarily been provided in animal models [3–5]. It is interesting to note that when considering the upper extremity (Fig. 1a) [6] or the lower extremity (Fig. 1b) [7] these architectural properties provide a first approximation to the peak performance capability of a skeletal muscle. Indeed such data are integral to virtually all of modern modeling software.

It has recently been shown that these basic architectural properties are excellent predictors of the functional properties of a whole muscle. For example, Winters, et al. [5] demonstrated that, for a very large rabbit skeletal muscle such as the tibialis anterior (TA), extensor digitorum longus (EDL), or second digital toe extensor (EDII), muscle properties can be predicted based only on the knowledge of the fiber length and the dimensions of the myofilaments within the sarcomeres [8]. This surprising result suggests that, to a first approximation for normal skeletal muscle, whole muscles can be treated as a scaled version of a sarcomere. It should be noted that this does not fit for muscle that has been tenotomized [9] or has become fibrotic [10].

**Fig. 2:**
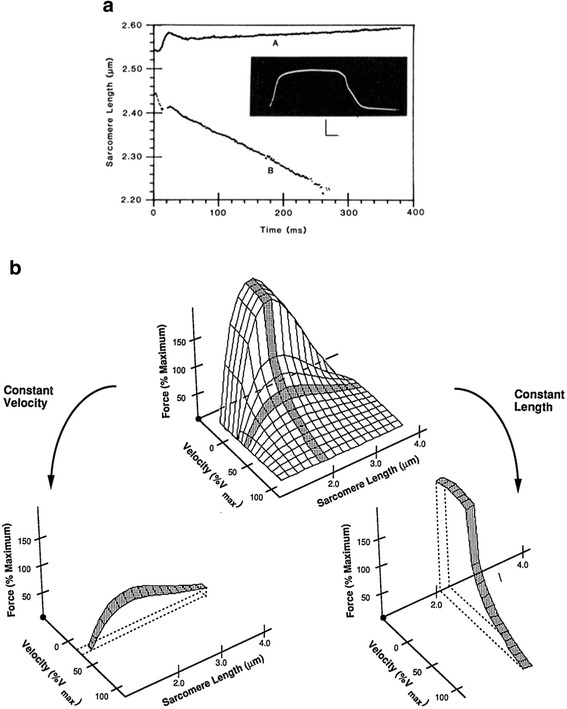
**a** Sarcomere dynamics of the center (trace A) and end regions (trace B) of a single skeletal muscle fiber. Fiber length = 5.47 mm . Center region = 2.03 mm from right tendon. End region = 0.32 mm from right tendon. Temperature = 12 °C Calibration bars for tension record: horizontal = 100 ms, vertical = 50 mg. (From: [13]. **b** The hypothetical muscle length-force-velocity surface for skeletal muscle. Shaded regions represent a “slice” of the surface at either constant length or velocity. A “slice” of the surface at constant length is simply a force-velocity curve measured at that length while a “slice” of the surface at constant velocity is simply a length-tension curve measured at that velocity (compare with Figs. 2, 3, 4). (From: [127])

**Fig. 3:**
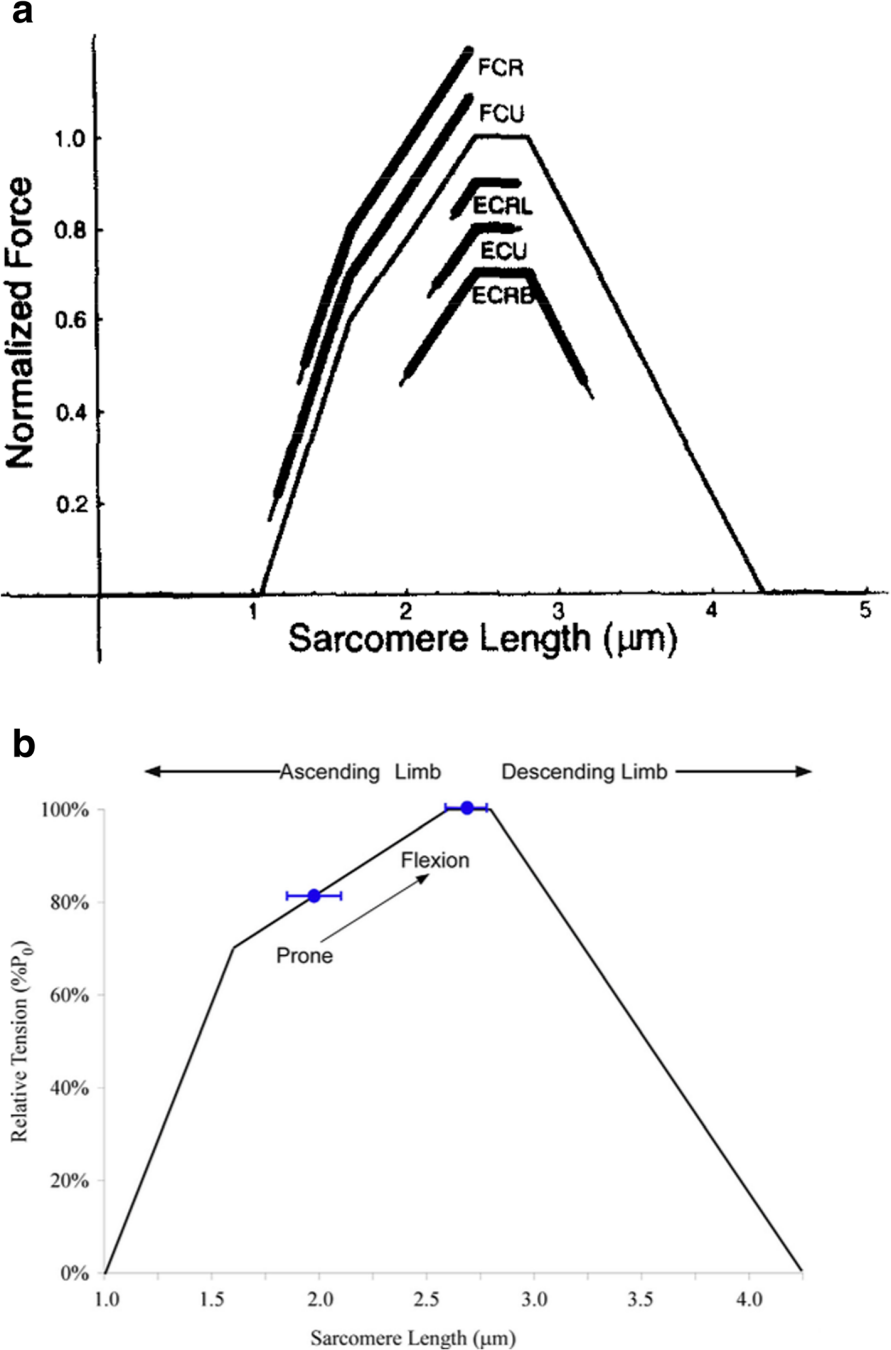
**a** Operating ranges of the wrist motors on the isometric sarcomere length-tension relation, Extensors operated primarily on the plateau region while the flexors operated predominantly along the ascending and steep ascending limbs. Lines at end of shaded areas represent the standard error for the data set. (Data presented for flexion-extension in neutral forearm rotation.) Mean sarcomere operating ranges were determined independently of the ensemble average muscle force- and torque-joint angle relations. Operating ranges when plotted against the isometric force-length relation may deviate slightly from the ensemble average force-joint angle relations. (From: [15]). **b** Sarcomere length operating range of the multifidus plotted on the human skeletal muscle sarcomere length tension curve (black line). Blue circles represent average sarcomere length obtained via biopsy in prone (*n* = 8) or lumbar flexion (*n* = 5). These data demonstrate that the multifidus muscle operates on the ascending limb of the length tension curve and becomes intrinsically stronger as the spine in flexed (arrow). (From: [16])

The sarcomere length tension controversy, which was at its peak during the 1970s and 1980s was based on the fascinating length tension experiments performed by Gordon Huxley and Julian [11], in which they showed that the dynamics of sarcomere properties along the length of a single cell are complex. However, these properties were inferred based on length measurements using a spot follower device that used foil clips upon a single fiber. Subsequent to those experiments, Lieber and Baskin [12, 13] directly demonstrated in a single cell that sarcomere length in the middle of the cell was qualitatively different than sarcomere length at the end of the cell (Fig. 2a). Note that the end sarcomere region shortened while the center sarcomere region lengthened. This phenomenon does not disrupt the cell because sarcomeres with negative velocities (lengthening) have enhanced force while sarcomeres with positive velocities (shortening) have diminished force. In fact, there are almost an infinite combination of lengths and velocities that sarcomeres can attain in a single cell. Thus, the proper way to think of isolated single fiber mechanics is that there exists a range of sarcomere lengths and velocities throughout the fiber, but these combinations are constrained to satisfy a constant force requirement across the fiber length (Fig. 2b). The observations that long sarcomeres within a single cell tend to be stretched by shorter sarcomeres within the same cell represented a mechanical instability and let investigators led investigators not to expect sarcomere lengths to occur on the descending limb of the length-tension relationship during any type of movement in vivo. This is one reason it was so surprising when Lieber et al. [14] reported that, using inter-operative laser diffraction in human arm muscles, sarcomeres in the extensor carpi radialis brevis (ECRB) could easily be situated on the descending limb of the length-tension curve. In fact, subsequent studies such as these in humans have demonstrated that a wide range of sarcomere lengths on the descending limb is possible (Fig. 3a). For example, the ECRB and the ECRL have sarcomere lengths that tend to be on the descending limb, while the wrist flexors such as flexor carpi ulnaris (FCU) and flexor carpi radialis (FCR) have sarcomere lengths on the ascending limb of the length tension curve [15]. This wide range in sarcomere lengths measured across muscles demonstrates that there is no single sarcomere length range that can be used to explain all muscles but that the function of a muscle will partially determine the sarcomere length range. For example, in the human wrist, it appears that wrist joint torque balance is the key design constraint [15]. In contrast, in the lumbar extensor muscle, specifically the multifidus, using intra-operative measurements of muscle properties, Ward et al. [16, 17] showed that lumbar extensors operate on the ascending limb of the length tension curve (Fig. 3b) so that, when the spine is flexed, sarcomeres are longer and stronger compared to when the spine is extended. This appears to be a model in which spine stability is maintained by altering the force with which sarcomeres can operate as a function of spine flexion angle. Peak restorative force occurs when it is most needed.

### Sarcomere length plasticity

**Fig. 4:**
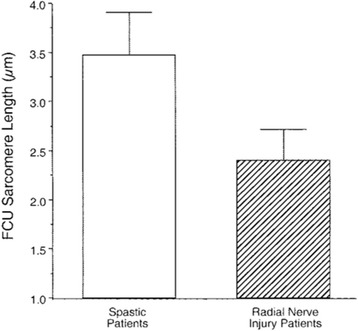
FCU sarcomere length measured with the wrist fully flexed in patients with spastic wrist flexion contractures (open bars, *n* = 6) or radial nerve injury (hatched bars, *n* = 12). Data represent mean ± SEM for each group. Sarcomere lengths between groups were significantly different as demonstrated by one-way ANOVA (*p* < 0.001). (From: [20])

In spite of the fact that sarcomere lengths have a preferred operating range, it is known that skeletal muscle is extremely plastic. That is, it can adapt to disease or altered use patterns. One of the most striking examples of this altered sarcomere length operative range is in children suffering from cerebral palsy (CP). In cases where the CP is severe, muscles are so affected that they actually entered into a contracted state called “contracture” in which a joint may end up in a fixed position. These joint contractures are extremely difficult to treat [18]. Potential treatments include splinting, casting and range of motion exercises even including electrical stimulation and, should the contracture not be relieved conservatively, injection with botulinum toxin or even surgery is indicated [19]. Given that the muscles from contractures are extremely short and under tension, we were surprised to find that the sarcomere lengths of contracture muscles are dramatically longer than the sarcomere lengths from typically developing control children (Fig. 4) [20]. In fact, this is unprecedented. There is no animal model in which either a muscle, a peripheral nerve or a central nervous system injury causes extremely long sarcomere lengths. The mechanism by which this occurs is not known. It is also interesting to note that in children with CP [21], the quantity of muscle stem cells [22] in contracture muscles is also highly depleted [21]. What is the relationship between depleted satellite cells and contracture in altered sarcomere length range? This is not known.

Unfortunately, the “party line” for sarcomere adaptation, which is still widely quoted, is when muscle length is chronically altered sarcomere length will simply adjust to that new muscle length. This concept is based on the results of William and Goldspink who published a series of excellent papers in the 1970s showing that in a normal rat, cat and mouse muscle that when a soleus muscle is immobilized in either a short length or a long length, that immobilization becomes its optimal length [23–25]. This concept has infiltrated the surgical literature and many surgeons believe that after surgery, muscle will simply reoptimize. Is this true? There is very little data to support this idea. However, in one study Boakes et al. [26] measured sarcomere length in a young woman who fractured her femur in the growth plate thus stunting her growth. The llizarov frame was placed on her femur (and her leg was lengthened approximately 9 cm. During this time period her sarcomere length changed from 2.6 μm to 3.11 μm. Given that her fiber length changed from 9.1 cm to 18.2 cm, this reveals a sarcomere length increase from 34,470 serial sarcomeres to 58,521 serial sarcomeres. Depending on the timing with which these sarcomeres were added, this reveals a sarcomere number increase of between 500 and 2000 serial sarcomeres per week. This is an astounding adaptation! Whether or not this is true for upper extremity muscles or even lower extremity muscles is not clear. In fact, in the tendon transfer literature [27] it is widely stated that a transfer can be put in at a high tension but should never be put in at a low tension. Based on these observations, it is not clear that upper extremity and lower extremity muscles adapt according to the same criteria.

**Fig. 5:**
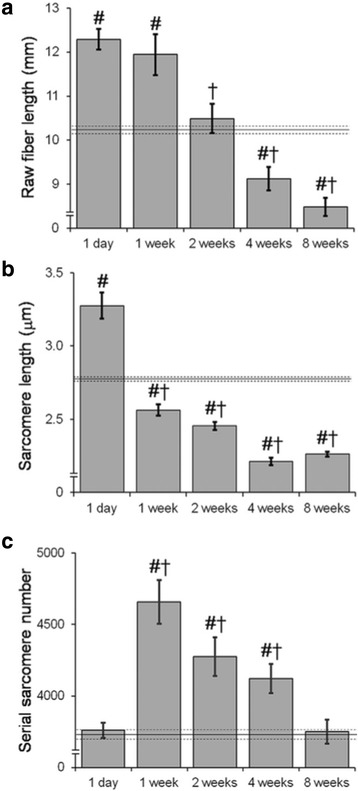
Raw fiber length (**a**), sarcomere length (LS, **b**) and serial sarcomere number (**c**) of the transferred muscle over the course of eight weeks after the operation. Horizontal solid and dashed lines represent the grand mean and SEMs for all the control muscle values, respectively. #; significantly (*p* < 0.05) different compared to the corresponding control muscle. †; significantly (*p* < 0.05) different compared to 1-day group. (From: [28])

For the sake of completeness and to underscore the fact that the rules of sarcomere number adaption are not known, an animal study by Takahashi et al., [28] should be mentioned. In this study the EDII of the rabbit was simply sutured to the extensor retinaculum to the point where it did not even cross a joint. At the time of suturing the sarcomere length was measured at 3.7 μm and then fiber length (Fig. 5a) and sarcomere length adaption were measured over time (Fig. 5b). Interestingly, sarcomere length started out at a long length but over 8 weeks decreased back to the starting sarcomere length. Initially it was believed that this was simply a similar situation as the Williams and Goldspink story above in that sarcomere length reoptimized to the length at which it was immobilized. However, when sarcomere number was actually measured it was shown that sarcomere number transiently increased and then decreased back to the original value (Fig. 5c). How can this be true? How can sarcomere length increase over one week and then for the next 8 weeks decrease back to the original value? The answer lies in the adaptation of the tendon. During this 8-week period the tendon increased in length and therefore the sarcomere length no longer was required to be longer. This very confusing experiment, the mechanism of which is not understood, points out the fact that muscle tendon adaption rules are simply not fully known. Of course, this result has important clinical implications for surgical procedures and rehabilitation protocols involving muscle-tendon units. It cannot be assumed, for example, that when the muscle-tendon unit length is increased chronically, that the added length will be adapted to by either the muscle or the tendon.

In summary, especially in the context of the neuromuscular science presented at the BANCOM conference, we concluded that the nervous system really “does not care much” about specific muscle force generating properties. It does not “micro-manage” the function required. The nervous system relies on the muscle to have characteristics consistent with a particular function and then simply controls relative timings of the various muscle activation patterns. Both the sarcomere length operating range and architecture appear to be “built-in” to individual skeletal muscles based on functional demands. Muscles are smart also in ways that we do not completely understand. The ability for serial sarcomere number to adapt in chronic altered use models, as well as in diseases such as CP is a completely new concept that has yet to be understood at the mechanistic level.

## Elastic mechanisms and muscle function

Muscle is a hierarchical structure, composed of a core of force generating sarcomeres arranged in bundles of myofibrils, fibers and fascicles that together form a complete muscle. Fibers, fascicles and muscles are surrounded by a matrix of connective tissues. We might view these connective tissues as just packaging; the scaffold that organizes an array of actuators (muscle fibers) into an effective geometry and attaches muscle to bone. However, it is clear that the elastic properties of tendons influence the force, power, and energetics of contraction, and evidence is growing that the elastic properties of the connective tissue matrix within muscle also provide an important mechanical role. While the sarcomere is the ultimate source of muscle power, the mechanical output of muscle depends very much on the properties of extracellular tissues, their arrangement, and the flow of elastic strain energy through them.

**Fig. 6:**
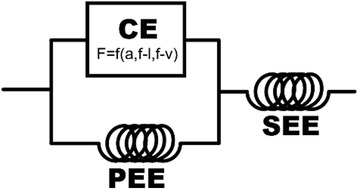
A Hill-type model of muscle with a contractile element (CE) arranged alongside a parallel elastic element (PEE) and in series with a series elastic element (SEE). Force development within the CE is a function of activation kinetics (a), force-length (f-l) properties, and force-velocity (f-v) properties. Force developed by the PEE depends on the CE length, while force in the SEE is equal to the sum of PEE and CE forces

The three element Hill-type model (Fig. 6) provides the simplest and arguably the most widely implemented model of muscle function that can characterize interaction between contractile and elastic elements [29, 30]. The model includes a contractile element (CE) that represents the fundamental mechanical behavior of the sarcomere, governed by activation kinetics, force-length properties, and force-velocity properties derived from isolated muscle studies. Springs in parallel with the CE and in series will influence the force, length and speed of the entire unit. This model is useful for exploring and describing the significance of the interaction between these different elements, and is commonly implemented in forward-dynamic simulations of movement [30, 31].

An obvious feature of the model as constructed is that the length change of the contractile element will differ from the length change of the muscle, because the series elastic element will change length whenever force varies. Muscle force output is strongly dependent upon CE length (force-length properties) and length change rate (force-velocity properties), so the series elastic element has the potential to significantly affect muscle performance. Studies of animal movement have demonstrated that the mechanical contributions of series elastic elements vary depending on the kind of movement.

In accelerative movements requiring significant mechanical power, series elastic elements can provide power outputs that exceed the capacity of the contractile elements. Such power amplification has been demonstrated for the simplest system, a muscle-tendon unit accelerating an inertial load [32, 33]. Frogs, bushbabies, and many arthropods amplify power via an elastic mechanism [34–37]. Power amplification by the SEE may be common; it has been observed in animals that are not specialized jumpers, such as in accelerating turkeys [38] (Roberts and Scales, 2002) and jumping humans [39].

In the same way that the series elastic component can amplify power by recoiling more quickly than it is stretched, it can also attenuate power input to the CE by stretching more quickly than it recoils. In situations where muscles must act as energy dissipaters rather than energy producers, the contractile element must be forcibly stretched, allowing the muscle to convert mechanical energy into heat. In jump landings, series elastic elements can reduce the rate of CE stretch and energy dissipation by initially absorbing energy during a rapid stretch, and then releasing that energy as the elastic element slowly recoils to stretch the CE [40, 41]. Muscle fibers and sarcomeres are prone to damage when forcibly stretched, thus the power-attenuating action of series elastic elements may provide a protective mechanism that reduces muscle damage.

Series elastic elements can also influence muscle function in cyclic movements. In walking, running, and hopping, the stretch and recoil of the SEE can accommodate the lengthen-shorten cycle of muscles associated with flexion-extension at joints. In fact, observations of different muscles in different organisms have found instances approaching the ideal, where length changes all occur in the SEE and the CE remains isometric [42, 43]. An open question remains: is this pattern beneficial? It has long been assumed that the stretch and recoil of elastic tendons reduces the cost of terrestrial locomotion because it reduces the work muscles must do to lift and reaccelerate the body with each step. However, a recent study of the energetics of force production in isolated muscles found that the cost of force production in a muscle undergoing cyclic stretch-shorten cycles is no greater than the cost of isometric force production [44]. This result calls into question the idea that replacing cyclic muscle work with cyclic series elastic work reduces the cost of locomotion. However, the stretch and recoil of tendons may allow muscles with short fibers to accommodate large joint excursions. Short fibered muscles produce force more economically, because a smaller volume of muscle is required to develop a unit force [42, 45].

### Springs in muscles and challenges to the concept of “parallel” elasticity

Since the inception of the Hill-type model, progress on understanding the many functional roles of series elastic elements has advanced significantly. Our current understanding of the mechanical role of parallel elastic elements is arguably much murkier. To start, it is unclear exactly what structure or structures are represented by the model’s parallel elastic element. When the model was formulated, physiologists pointed to the connective tissue matrix that envelops and organizes muscle fibers as the source of passive muscle tension and parallel elasticity [46, 47]. The collagenous matrix is elastic, organized in parallel with the contractile element, and thus an obvious potential source of parallel elasticity [48]. However, since the identification of titin in the late 1970’s it has been proposed that much of the passive tension in muscle results from this molecular spring. Titin runs from Z-line to Z-line and so is arranged in parallel with the contractile element, and it is highly elastic. If titin is the source of parallel elasticity, the Hill-type model of a simple spring in parallel with the contractile element would miss important properties of titin’s behavior, including Ca^2+^-mediated modulation of stiffness [49–51], and a possible direct interaction of titin and acto-myosin force production [52, 53]. The significance of these features of titin for muscle function is an active area of research. However, the relative significance of titin vs. connective tissue elements to passive stiffness and parallel elasticity in muscle remains controversial. The relative importance of titin vs. extracellur matrix for the passive spring-like properties of muscle is also still under debate, with some evidence favoring titin as the dominant source of parallel elasticity [54], while other studies indicate that connective tissue structures determine passive muscle stiffness [55, 56]. Resolving these questions is important to our understanding of the behavior and control of the muscle actuator in vivo.

A question motivating current research in my lab regards not where the passive elasticity in muscle resides, but whether the parallel arrangement of the Hill-type model accurately characterizes its mechanical role. The two-dimensional construction of the Hill-type model defines a geometric link between the contractile element and the PEE. According to the model, lengthening and therefore elastic loading of the PEE can occur only when the CE is lengthened. However, muscles operate in three-dimensions. Shortening of the contractile element is always accompanied by an expansion of the muscle fiber in the radial dimension, because muscles are essentially isovolumetric over the time course of a contraction [57]. Force production in muscle contractile elements often involves forces directed off-axis to the orientation of the contractile element, because in pennate muscles the contractile elements are oriented at an angle (the pennation angle) to the line of action of the muscle. We hypothesize that both of these features of muscle contraction result in the stretch of elastic elements that are arranged structurally in parallel with the force generating elements of muscle. This three-dimensional arrangement decouples the link between CE and “PEE” length implied by the Hill-type model, and thus loading of elastic tissues may occur under many circumstances that would not be predicted by the Hill-type model.

**Fig. 7:**
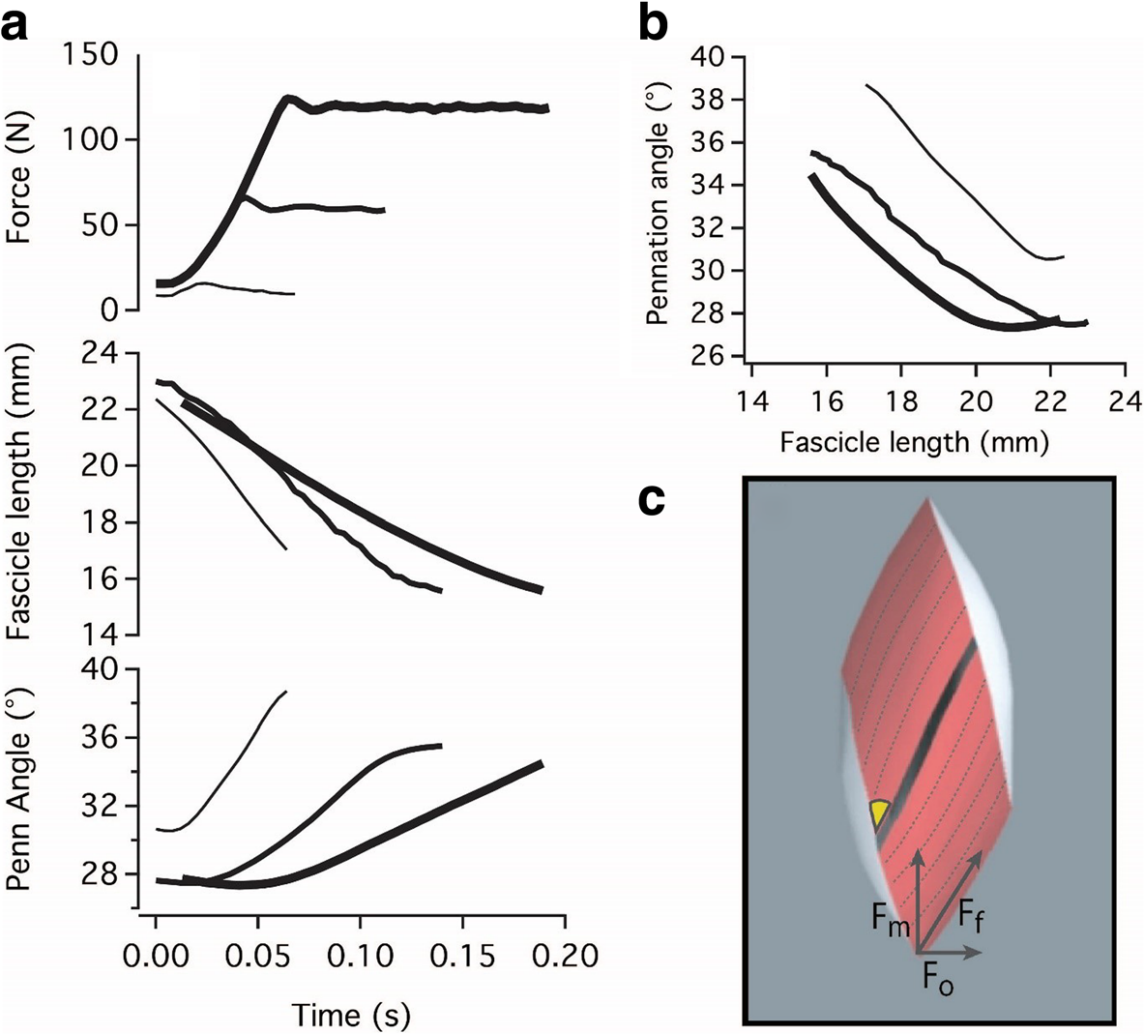
The relationship between pennation angle and muscle fiber length is not fixed for a given muscle, but varies according to contractile force. Three isotonic contractions (**a**) show a pattern of rotation of fibers that is proportional to force. Lower-force contractions involve more fiber rotation (**b**). It is hypothesized that the component of fiber force (F_f_) oriented orthogonal (F_o_) to the muscle force (F_m_) tends to compress the muscle and reduce fiber rotation (**c**). At lower forces, a low F_o_ allows for more fiber rotation to higher angles of pennation. Fiber length and pennation angle measurements were obtained via implanted radiopaque markers and biplanar fluoroscopy [128]

One potential role of “off-axis” loading of parallel elastic elements is in the determination of architectural gear ratio (AGR). AGR is defined as the ratio of shortening speed of the muscle to the shortening speed of the fibers, or contractile element [58]. In pennate muscles AGR is typically greater than one, because as fibers shorten they also rotate to greater angles of pennation, and this rotation contributes to muscle shortening (Fig. 7). A given muscle does not always operate with the same AGR. It has been observed in several muscles that AGR changes as a function of force, because the shape changes muscle undergo, and the amount of fiber rotation, varies as a function of force (Fig. 7; [44, 59, 60]. What determines the shape trajectory of a contracting muscle? We hypothesize that the interplay of the forces generated by the CE, fluid pressure within the muscle, and the elastic behavior of intramuscular connective tissues determines dynamic muscle architecture. The tendency of off-axis force to compress a muscle in one direction leads to the stretch of elastic elements in orthogonal directions, and this stretch (and deformation) is therefore dependent on muscle force (Fig. 7).

An implication of the hypothesized model of variable muscle shape change is that off-axis forces regularly load elastic elements in the muscle in a way that is not apparent, or possible, in the 2D construct shown in Fig. 6. Thus, elastic tissues within muscles may be stretched and contribute to the mechanics of contraction in many, possibly all, contractions of pennate muscles, independent of CE length. Our current focus is on the off-axis loading of the extracellular matrix (ECM), but the protein skeleton of the sarcomere and myofiber also include elastic structures oriented off-axis to the sarcomere (e.g., the protein disc arrays of the Z-lines [61], thus there are many structures that may contribute to elastic mechanisms involved in muscle shape changes.

An emergent theme over the course of the BANCOM meeting was the significance of the properties of the actuator for the control task faced by the nervous system. If we accept the notion that coordinated movement relies in part, or perhaps heavily, on the adaptive mechanical behavior of a contracting muscle (the “brains within the muscle”), we must consider that this behavior is determined in part, possibly in large part, by the behavior of the elastic tissues associated with muscles, and that this interaction may not be well-characterized by many existing models of the function of this hierarchical structure.

## Muscle has a mind of its own: a computational framework to model the complex process of muscle adaptation

As discussed in previous sections, muscle sarcomere properties, connective tissue properties, elastic elements, and architecture all influence muscle mechanics and function in unique interconnected ways. However, the reverse is true as well: the nature and degree of movement, intersected with the biochemical and cellular interactions within skeletal muscle, lead to adaptations in sarcomere properties, connective tissue properties, elastic elements, and architecture. For example, Rick Lieber’s section describes findings from individuals with cerebral palsy – sarcomere lengths are overstretched in children with muscle contractures, and Sabrina Lee’s section features findings from individuals with stroke – shear wave speed in passive muscle tissue, as measured by ultrasound elastrography, is increased in spastic muscles, as compared to healthy controls, suggesting an increase in passive stiffness of the muscle. Consideration of the feedback between movement and muscle adaptation is critical to understanding neuromuscular control and pathology. Often in the neuro-motor and biomechanics literature it is assumed that muscle adaptation (for example muscle contracture in spasticity) occurs as a biomechanically driven process responding to abnormal neural control; however, this assumption simplifies both the biomechanical and cellular signals that lead to muscle adaptation. We are developing a modeling framework for integrating biomechanical and cellular signals to predict muscle adaptation to use, disuse, injury, and neuromuscular disease.

The focus of my presentation at the BANCOM meeting was to describe the recent work from my group on the development of new models that predict a given muscle’s adaptive response to underuse, overuse, or disease. I titled my talk “Muscle has a mind of its own” to emphasize the point that muscles are organs with agency over their adaptive processes, and they are composed of many cell types that all interact to determine the adaptive response of the muscle. These cells do not have pre-programmed behaviors that are dictated by the nervous system so that they do what “makes sense” from the standpoint of human movement and energetics. Rather, the behaviors and responses of these cells are determined by sub-cellular signaling pathways that are influenced by both mechanical and biochemical stimuli. In this section, I will focus on the specific case of muscle atrophy, whether due to disuse, aging, or disease.

Muscle atrophy is caused by changes in the behaviors of the multitude of cells that make up muscle tissue, and these behaviors are affected by changes in, or the loss of, mechanical stimulation [62]. Muscle fiber protein production decreases in the absence of mechanical stimulation [63], and protein breakdown transiently increases then diminishes during disuse [63]; both of these responses are dependent on muscle fiber type [64]. Furthermore, many non-muscle fiber cells are also sensitive to the mechanical environment in muscle [65–67]. For example, fibroblasts are affected by changes in mechanical stimulation which alters their production of ECM [68], and affects their proliferation [69, 70], apoptosis [71], and growth factor secretion [72]. Growth factors, in turn, affect muscle fiber and fibroblast behaviors [73–76], and interactions between these cell types may affect ECM volume changes observed during muscle atrophy [77, 78]. While a wealth of experimental studies have captured these individual phenomena, the critical challenge is transforming all the information into a holistic understanding of how cell interactions dictate adaptive and atrophic processes in different muscles.

Historically, muscle models have focused on predicting and exploring the mechanics of force generation. Computational and mathematical models are used to predict muscle force generation at multiple biological scales, including cross-bridge dynamics [79, 80], sarcomere and half sarcomere dynamics [81, 82], muscle fiber recruitment [83], micro-scale fiber mechanics [84, 85], muscle tissue dynamics [86–88], as well as limb and body locomotion [89]. These models revealed important relationships between force production and skeletal muscle structure at individual length scales. However, the majority of published computational models of muscle fall short of predicting *how* muscle adapts to disuse or disease, and we have posited that the use of other computational modeling approaches, such as agent-based modeling (ABM), will enable deeper understanding of the interplay between biomechanics and biochemistry in the muscle adaptation process.

Agent-based computational modeling is emerging as a useful approach for studying and predicting how cell behaviors and cell interactions give rise to dynamic tissue-level responses, such as growth and adaptation [90]. Agent-based computational modeling has been used to understand how different tissues adapt to various physiological and pathophysiological stimuli. Agent-based computational models simulate the individual behaviors of biological cells, each of which is represented as an individual “agent” in the model. Because the dynamic behaviors of hundreds of thousands of agents, or cells, can be simulated within a heterogeneous tissue environment, agent-based computational models can predict tissue-level outcomes that result from the emergent and stochastic interactions between cells and their changing environment. Importantly, the “rules” that govern individual agent behaviors in agent-based computational models incorporate molecular-level mechanisms that have been derived from empirical studies. Hence, the rule set of the agent-based model constitutes a comprehensive integration of currently available data pulled into a framework that can reveal cause-and-effect relationships between mechanisms across different cell types and stimuli.

**Fig. 8:**
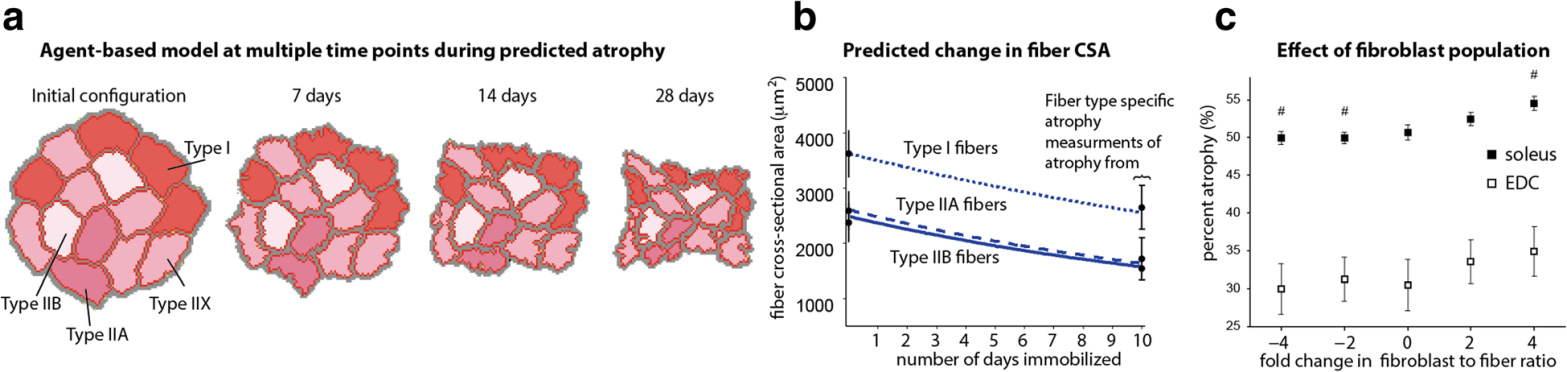
Agent-based simulations of muscle remodeling response to disuse-induced atrophy. The ABM predicts muscle atrophy over time (**a**) in a manner that was consistent with experimental measurements (**b**), and can be used to examine the effects of varying the populations of non-muscle cells that are found in muscle tissue, like fibroblasts (**c**), on muscle atrophy

Recently, we created what we believe was the first agent-based computational model of skeletal muscle (Fig. 8), and we have used it to study skeletal muscle atrophy in response to disuse [91]. We used our agent-based model to study the relationship between protein synthesis and degradation during atrophy and to explore how muscle tissue architectural features, such as muscle fiber type distribution and muscle fiber cross-sectional area distribution, influence the extent to which muscles atrophy. Our model incorporates secreted biochemical growth factors and chemokines that interact with both muscle fibers and fibroblasts to influence the process of atrophy over time. We used our computational model to simulate muscle-specific fiber architectures for 49 different rat muscles, and the model predicted decreases in muscle fiber size that were consistent with published experiments in rats. Analysis of these simulations revealed that both muscle fiber type and muscle fiber size distributions influenced reductions in muscle mass, even though no single tissue architecture parameter could predict muscle atrophy rate.

Since the initial publication of our agent-based computational model [91], we have adapted it to the prediction of muscle’s response to injury and regeneration [92]. The updated agent-based model simulates the effects of inflammation on muscle adaptation and regeneration. For example, one prediction from our agent-based model is that augmenting the recruitment of monocyte-derived macrophages via pharmacological administration of macrophage colony stimulating factor (M-CSF) shortly after muscle injury may accelerate muscle regeneration via mechanisms that involve satellite stem behaviors. We have now validated the model’s prediction by performing an independent set of experiments in rats [93]. Specifically, by performing laceration injuries in the rat tibialis anterior, we have now experimentally confirmed the agent-based model’s prediction that priming injured muscle with M-CSF accelerates muscle regeneration relative to vehicle control.

Muscle adaptation modeling has a broad range of applications. We are currently improving the predictive power of the adaptation-modeling framework through linking the agent-based models with micro-mechanical [85] and/or macro-mechanical [94] computational models. These coupled models could provide mechanistic information about how biomechanical influences affect (and are affected by) biochemical influences (i.e. mechanisms of feedback between biomechanical and biochemical signals). With these two advances, multi-scale modeling will enable the prediction of functional muscle adaptations, as well as facilitate incorporation of behaviors related to cell mechano-sensitivity. Overall, all of these efforts will enable rational design of therapeutic interventions that exploits the complex remodeling processes within skeletal muscle to maximize functional recovery from a wide variety of neuromuscular conditions.

## Muscle properties of spastic muscle (stroke and CP)

When considering how motor performance is impaired in individuals with decreased mobility and movements, altered force generation, in both timing and amplitude, is often identified as a primary contributor. Properties such as architecture (e.g. fascicle and sarcomere length, pennation angle) and material properties affect the force-length and force-velocity relationships of muscle, as well as force transmission. A question that is raised repeatedly is whether spastic muscle of individuals with neurological impairments such as stroke and cerebral palsy (CP) are inherently physiologically healthy and changes are due to adaptations to altered neural control, or does spastic muscle have intrinsic pathological characteristics. Knowing if certain muscle properties change and how they change can provide crucial information for clinicians to make decisions related to diagnosis and rehabilitative treatment planning. This section will give a brief summary of what is currently known about muscle properties in these populations, but will primarily focus on material properties such as muscle stiffness.

There has been much work investigating changes in muscle architectures in individuals with spastic muscles. These architectural parameters include: muscle size, muscle belly length, fascicle length, sarcomere length, and pennation angle. It is undisputed that there is a loss of muscle mass in stroke survivors and individuals with CP which directly impacts the amount of force that can be generated. There is simply a loss of contractile tissue. This has typically been measured as decreased volume and cross-sectional area using MRI [95, 96] and ultrasound [97, 98] and decreased muscle thickness using ultrasound [99]. The muscle belly length has also been consistently measured to be shorter [97, 100, 101]. In the past 15 years or so, there has been much use of ultrasound to measure muscle architecture at the fascicle level with measures of fascicle length and pennation angle. Stroke survivors have been reported to have shorter fascicles of muscle of the paretic side compared to the non-paretic side and non-impaired control individuals [102–104] (8–10). However, in CP, it is inconclusive whether there are changes in fascicle length as several studies report conflicting results [97, 98, 100, 105–108]. One explanation for this inconsistency is related to differences in sarcomere length; two fascicles can be of the same length if one has less sarcomeres but longer sarcomeres. The development of intraoperative laser diffraction to measure sarcomere lengths has provided great insight into how these basic contractile units are affected in spastic muscle. In CP, sarcomeres have been found to be longer in upper [20] and lower extremity muscles [55]. Lieber et al. report longer sarcomere lengths in wrist flexors of children with chronic wrist flexion contractures compared to patients with radial nerve injury. Although the wrist was in a constant flexed position and thus, the overall muscle was at a shorter length at rest, the sarcomeres were found to be longer [20]. Thus, interpretation of fascicle length differences must be made with caution as sarcomeres are the basic contractile units and directly impact the force-length and force-velocity relationships, and thus, muscle mechanics and function. A challenge of measuring sarcomere lengths in vivo is the invasive nature of the intraoperative laser diffraction method such that little is known about sarcomere length in stroke survivors and other clinical populations. Current emergent technologies such as minimally-invasive optical microendoscopy [109, 110] will hopefully provide insight into sarcomere lengths in populations where intraoperative laser diffraction is not possible.

Another explanation of the inconsistent fascicle length results is that many reports include fascicle lengths measured only at one angle such that comparisons may be between muscles that are at different lengths. Thus, reporting strain over range of motion or over a dynamic task is more informative. Zhang et al. reported shorter fascicle lengths of the medial gastrocnemius in stroke survivors across the range of motion, but also estimated the force length curve for which they found to be shifted to the left and was more narrow on the paretic side compared to the non-paretic side [111]. This suggests that fascicles may operate more on the ascending and descending limb over the range of motion and thus, generating less force. Evidence for changes in pennation angle in spastic muscle is also inconsistent between studies [98, 105–108].

In addition to altered force generating capacity, increased joint and muscle stiffness likely contribute to decreased range of motion and impairment movement. Many studies have reported increased stiffness at the joint level [111–115] and estimates of muscle stiffness [102, 111, 116] in individuals with spastic muscle. Measuring stiffness and determining and distinguishing the contributions is challenging. By combining dynamometry, electromyography, and electrical nerve stimulation, estimates of passive stiffness, neutrally-mediated stiffness, and active stiffness can be calculated. Passive stiffness can be attributed to non-contractile tissue such as tendons, ligaments, connective tissue and other soft tissues, though often, muscle passive stiffness is considered the primary contributor. A primary source of passive stiffness in muscle has been attributed to the extracellular matrix as the modulus of fiber bundles was five times higher than fiber groups which do not contain extracellular matrix and single muscle fibers [117]. Neurally-mediated reflex stiffness may be altered due to descending influences on the monosynaptic reflex between muscle spinal afferents and the alpha-motor neurons. Sources of active stiffness include the cross-bridges of the actin and myosin filaments. Sinkjaer et al. demonstrated that total stiffness in the spastic leg of stroke survivors was greater than that measured in the contralateral non-paretic leg [112]. Passive stiffness accounted for almost all of difference between total stiffness of the paretic and non-paretic leg. Interestingly, reflex stiffness was not significantly different between the two limbs. Gao and Zhang reported increased muscle stiffness in the medial gastrocnemius muscle of the paretic limb of stroke survivors compared to that of healthy control subjects [111]. They estimated passive muscle stiffness by calculating the slope of estimated muscle force and fascicle length. Gastrocnemius muscle force was estimated by dividing ankle torque by Achilles tendon moment arm. Although this approach has many assumptions and does not provide direct quantification of muscle stiffness, it does use fascicle strain rather than simply joint angle for calculation of stiffness which is often used [118, 119]. Quantifying muscle stiffness has challenges and current methods have crucial limitations. For example, there is much work accomplished on quantifying joint stiffness, but that measurement encompasses the joint capsule, all the surrounding ligaments, and all muscle that cross the joint. There are estimates of muscle stiffness using joint torque and moment arm calculations, but accurate stiffness measurements of individual muscles have not been possible other than in situ measurements made in animal experiments. A recent novel method that provides more accurate estimates of muscle stiffness is using tendon indentation where musculotendon stiffness is estimated from the force and displacement measured during tendon indentation in stroke survivors [115]. Chardon et al. (2010) reported that passive stiffness was increased in the biceps brachii on the paretic limb compared to the non-paretic limb [115].

One of the main challenges with choosing the optimal patient-specific intervention, in particular with individuals who have had a stroke, is to due limited clinical tests to distinguish between passive and active stiffness. The ability of clinicians to do so is imperative as a treatment, like botulinum toxin, would greatly help an individual whose lack of mobility is largely contributed by or influenced by altered muscle innervation or on a smaller scale, recruitment of motor units. However, an individual who has increased passive stiffness due to increased connective tissue or changes in the extracellular matrix may not benefit as much with botulinum toxin-A injection.

Shear wave ultrasound elastography, in particular SuperSonic Imagine (SSI) has been used for quantifying muscles stiffness with success. These techniques builds on traditional elastography and overcomes several important limitations of earlier methods to evaluate muscle material properties, specifically stiffness. This technique is based on the relationship between shear wave velocity and shear modulus such that shear waves will propagate faster in tissues that are stiffer. Using the transducer to both induce and track shear wave propagation, shear wave velocity is calculated for a specified region of interest. Several studies have investigated the stiffness of muscles using SW elastography in healthy adults [120–122] and in clinical populations [123–126]. Increases on average of 60% in SW velocity in the passive biceps brachii muscle of the paretic side have been reported compared to the non-paretic side in individuals who have had a stroke and to healthy control indicating increased stiffness. These measures were also strongly correlated with increased echogenicity [123], a measure from B-mode ultrasound which can indicate differences in muscle composition, suggesting that muscle stiffness may be influenced by changes in muscle composition. However, little is known about the changes in muscle composition in stroke-impaired muscle, as well as the relationship between changes in muscle composition and mechanical properties of muscle. In children with hemiplegic CP, shear wave velocity was greater in the gastrocnemius muscle of the more-affected side than the less-affected side [123–126]. These examples demonstrate the potential for shear wave elastography for providing a quantitative measure of altered muscle material properties that can differentiate impairment between sides in hemiplegia.

A reoccurring theme that was discussed during the BANCOM muscle session was that, although we know details in changes such as sarcomere and fascicle length, muscle volume and mass, there is a paucity of details regarding the interactions between intracellular proteins and the surrounding tissue that can occur from the cellular level to whole muscle and little is known about the sequence of events that occur post-neurological injury. There are also confounding changes related to aging and development that are difficult to account for clinical populations such as stroke and cerebral palsy, respectively.

## Conclusions

In conclusion, from the level of sarcomeres to whole muscle, muscle properties are altered in spastic muscles. Our knowledge of muscle properties in clinical populations such as stroke and CP is constantly growing as more accurate and non-invasive tools are constantly emerging. Although there is much unknown about the pathophysiological mechanisms of spasticity, advancements in motor control and muscular measurements will allow investigation of the interactions between the nervous and muscular systems to provide clarity to the question of whether and how muscle adapts to altered motor control and possible pathologic intrinsic muscle properties that are independent to changes in the nervous system.
